# Marine Drugs Regulating Apoptosis Induced by Tumor Necrosis Factor-Related Apoptosis-Inducing Ligand (TRAIL)

**DOI:** 10.3390/md13116884

**Published:** 2015-11-13

**Authors:** Mohammed I. Y. Elmallah, Olivier Micheau

**Affiliations:** 1 Department of Chemistry, Faculty of Science, Helwan University, Ain Helwan, Cairo 11790, Egypt; E-Mail: mohamed_almallah@science.helwan.edu.eg; 2 Institut National de la Santé et de la Recherche Médicale, UMR866, Dijon F-21079, France; 3 Unité de Formation et de Recherche, Science de Santé, Dijon F-21079, France

**Keywords:** TNF, TRAIL, Apoptosis, resistance, cancer, marine drugs, alkaloids, carotenoids, sesquiterpenes, macrolactones

## Abstract

Marine biomass diversity is a tremendous source of potential anticancer compounds. Several natural marine products have been described to restore tumor cell sensitivity to TNF-related apoptosis inducing ligand (TRAIL)-induced cell death. TRAIL is involved during tumor immune surveillance. Its selectivity for cancer cells has attracted much attention in oncology. This review aims at discussing the main mechanisms by which TRAIL signaling is regulated and presenting how marine bioactive compounds have been found, so far, to overcome TRAIL resistance in tumor cells.

## 1. Introduction

Hallmark features of cancer include, among others, genetic mutations that regulate normal cell homeostasis. Mutations of tumor suppressor genes and oncogenes lead to alterations of cell proliferation and cell death pathways, contributing to cancer progression and resistance to treatments [1,2]. Proteins encoded by these master genes play a central role in the occurrence, development, and stability of an organism [2], particularly during embryonic development and immune surveillance [3]. Apoptosis is a physiological cell death process characterized by chromosomal DNA fragmentation, nuclear disintegration, cell shrinkage, translocation of the phosphatidyl serine moiety to the outer membrane leaflet, and membrane blebbing [4]. Impairment or resistance to apoptosis leads to various diseases, including autoimmune diseases, degenerative disorders, and cancer. The molecular mechanisms by which tumor cells gain resistance to apoptosis mainly include (1) a dysregulation of the mitochondrial pathway; (2) the inactivation or the loss of caspases; and/or (3) a deficiency of death signals through the transmembrane death receptors of the TNF superfamily [5,6,7]. Overcoming resistance to apoptosis is probably the most important challenge in oncology. This review focuses on TRAIL signaling regulation by marine bioactive compounds, as it is anticipated that these combinations may be valuable in cancer therapy.

## 2. TRAIL-Signaling and Regulation in Cancer

### 2.1. Activation of Apoptosis by TRAIL

**Figure 1:**
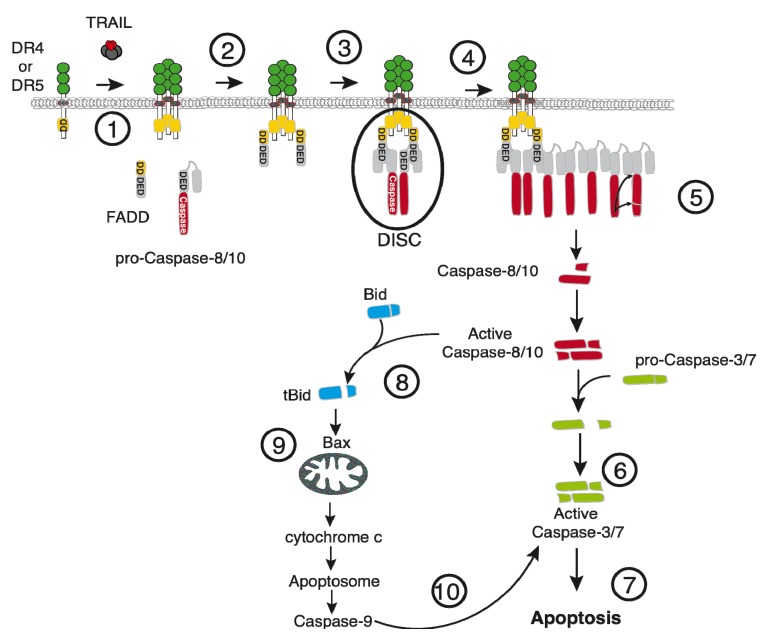
Schematic representation of apoptosis induced by TRAIL. Binding of TRAIL to its agonistic receptors (DR4 and/or DR5) leads to their oligomerization (1), and the subsequent recruitment of the cytosolic adapter protein FADD through DD homotypic interactions (2). FADD, in turn, enables recruitment of the initial pro-caspase-8/-10 through DED interactions leading to the formation of the so-called DISC (death-inducing signaling complex (3)). Within the DISC, chains of caspase-8/-10 are assembled (4), allowing self-processing of caspase-8/10 and release of their active fragments to the cytosol (5), where they activate, by proteolytic cleavage, effector caspases (6) to execute the apoptotic program (7). Amplification of the signal through the intrinsic pathway is sometimes required when caspase-8 is not sufficiently activated. In this scenario activation of mitochondria is induced by caspase-8/-10 through cleavage of the BH3-only Bcl-2 family protein Bid (8), whose truncated version (tBid) is able to translocate to mitochondria and induce a change of their outer membrane permeability, through Bak and Bax interactions (9), allowing cytochome c release, activation of caspase-9, another initiator caspase able to activate the executioner caspase-3/-7 (10).

TRAIL agonist receptors contain an intracellular death domain (DD) that enables the assembly of the death-inducing signaling complex (DISC), leading eventually to apoptosis (Figure 1). Like most death ligands of the TNF superfamily, TRAIL binding to its cognate agonistic receptors triggers their aggregation, and with the exception of TNF-R1 [8], subsequent recruitment of the adaptor protein FADD (Fas Associated Death Domain) at the membrane level [9], through DD homotypic interactions (Figure 1). Once associated with TRAIL agonist receptors, FADD in turn enables the recruitment of the initiators caspases, namely caspase-8 and/or caspase-10, through its death effector domain (DED). DED-containing proteins, like proteins harboring DD’s, are able to interact with themselves and one another [10]. They are required for DISC assembly. Although the precise stochiometry of this complex remains partially unsolved, two independent studies have recently demonstrated that caspase-8 recruitment could lead to the formation of caspase-8 chains, representing up to nine-fold more caspase-8 than FADD [11,12]. Recruitment of these initiator caspases in a defined subcellular compartment within this scaffold is sufficient to induce their activation. An induced-proximity model was originally proposed in the late 1990’s to explain caspase-8 processing and activation following stimulation with another ligand of the TNF family, namely CD95L/Fas ligand [13,14]. Within the DISC, self-processing of caspase-8 or caspase-10, leads to the release of their active fragments to the cytosol. These fragments are composed of a large and a small catalytic subunit. Their assembly is sufficient to induce the cleavage and the activation of executioner caspases, including caspase-3 or caspase-7, leading to apoptosis (Figure 1).

The contribution of caspase-8 in triggering apoptosis upon engagement of the death receptors is largely accepted in the community. However, the ability of caspase-10 to substitute for a lack of caspase-8 remains a matter of debate [15], despite the fact that this initiator caspase was shown to restore both TRAIL- and Fas ligand-induced apoptosis in caspase-8-deficient neuroblastoma [16] and the caspase-8-deficient Jurkat T lymphoma derivative cell line [17]. Irrespective of whether caspase-10 is able to substitute, or not, for caspase-8, the outcome of their activation, upon TRAIL stimulation, heavily relies on the cell type which, itself, is dependent on the strength of activation of these initiator caspases. In so-called type I cells, caspase-8/-10 activation is sufficient to trigger apoptosis directly through executioner caspases, while activation of executioner caspases and apoptosis in type II cells require mitochondria (Figure 1). Activation of this intrinsic pathway is mediated through the cleavage of Bid, a caspase-8 and -10 substrate, belonging to the BH3-only Bcl-2 family [18,19]. Once cleaved, truncated Bid (tBid) translocates to mitochondria and induces mitochondrial outer membrane permeabilization, through oligomerization of Bak and/or Bax, allowing the release of cytochrome c to the cytosol [20,21] and formation of the apoptosome [22]. This soluble multimeric complex scaffold, composed of the multimeric adaptor protein APAF-1, cytochrome c, dATP, and caspase-9 enables the activation of the initiator caspase [23]. Once activated, caspase-9 cleaves and activates executioner caspases.

### 2.2. Negative Regulators of TRAIL

**Figure 2:**
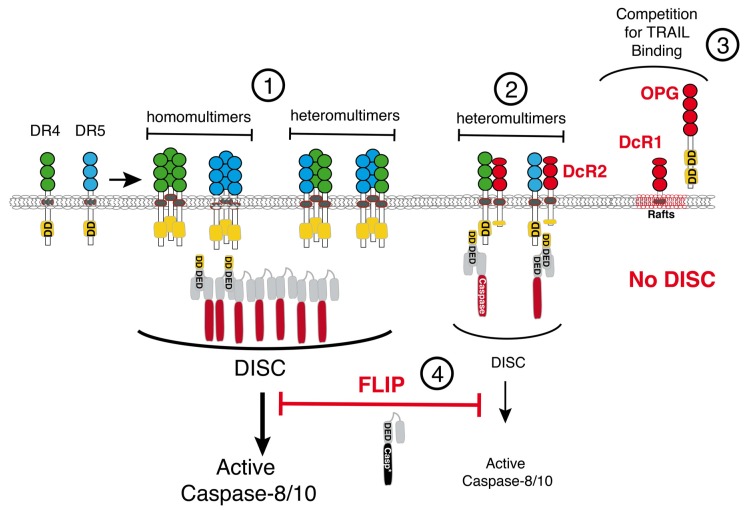
Schematic representation of TRAIL pro-apoptotic proximal signaling regulation. Apoptosis induced by TRAIL is initiated through homo/or hetero-oligomerization of DR4 and/or DR5 and subsequent chain assembly of initiator caspases (1). Proximal activation of caspase-8 by these receptors can be inhibited by TRAIL antagonistic receptors DcR2 (2), DcR1, and OPG (3), as well as by the cellular caspase-8 inhibitor, c-FLIP (4) (see text for explanations).

Efficient activation of initiator caspases, upon TRAIL stimulation, is tightly dependent on the quality of the DISC, determining the strength of activation of the initiator caspases [24]. Irrespective of whether the apoptotic signal is transduced by DR4 or DR5, through homomultimers or heteromultimers, and provided that enough receptors, FADD, or initiator caspases are expressed by a given cell line, selective inhibition of this pro-apoptotic signaling pathway can result from concomitant expression of any of the three antagonistic receptors, namely DcR1 (TRAIL-R3) [25,26], DcR2 (TRAIL-R4) [27,28], or a soluble receptor called OPG (osteoprotegerin) [29] (Figure 2). Amongst these antagonist receptors, OPG is probably the weakest inhibitor, owing to its lower affinity for TRAIL [30]. OPG exhibits much higher affinity for RANKL and its physiological function has, so far, mainly been ascribed to osteoclasts development and activation [31]. Nevertheless this soluble receptor was shown to be able to compete, to some extent, with DR4 or DR5 for TRAIL binding [29,32]. DcR1 and DcR2, on the other hand, owing to their higher affinity for TRAIL [26,30], have been studied more extensively to assess their ability to inhibit TRAIL pro-apoptotic signaling. These receptors are both devoid of functional DD and display differential inhibitory properties. While DcR1, whose c-terminus harbors a glycosylphosphatidyl-inositol (GPI) motif, is localized in lipid rafts, DR4, DR5, and DcR2 are mostly found in non-lipid rafts portions of the plasma membrane, even when engaged within the TRAIL DISC [33]. DcR1 is unable to form heteromultimers with DR4 or DR5 upon TRAIL stimulation. Therefore, like OPG, DcR1 appears to inhibit TRAIL-induced apoptosis in a competitive manner by titrating its cognate ligand [33]. DcR2, on the other hand, is co-recruited with DR4 [34] and DR5 [33] within the TRAIL DISC, in non-lipid rafts fractions (Figure 2). Recruitment of DcR2 within the TRAIL DISC leads to the formation of a heteromultimeric complex, whose ability to trigger initiator caspase activation is reduced, as compared to DR4 and/or DR5 homomultimeric or heteromultimeric complexes [33,34]. Since DcR2 is devoid of functional DD, it has been proposed that its recruitment within the TRAIL complex may impede caspase-8 activation by mere steric hindrance [35]. More recently, DcR1 and DcR2 were found to be able to act, in addition, in trans by titrating TRAIL on stroma cells within the tumor microenvironnement [36].

**Figure 3:**
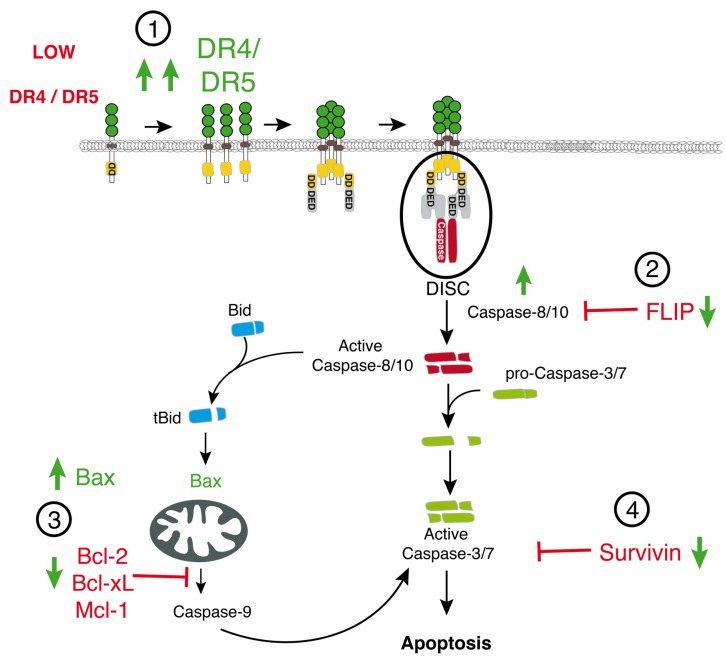
Schematic representation of the main TRAIL signaling or regulator components targeted by conventional chemotherapies to restore TRAIL-induced apoptosis. Restoration of apoptosis induced by TRAIL in resistant tumor cells can be induced through restoration of DR4 and/or DR5 (1), caspase-8/10 (2), or Bax expression (3), or down-regulation of anti-apoptotic factors such as c-FLIP (2), Bcl-2 family proteins (Bcl-2, Mcl-1, or Bcl-xL (3)), and/or survivin or XIAP (4).

In addition to these non-functional receptors, or the lack of expression of the agonistic receptors DR4 or DR5 [37,38,39], resistance to TRAIL-induced cell death can arise in tumor cells owing to a large variety of events, including a loss of caspase-8 expression [40] or the overexpression of c-FLIP [41,42] (Figure 2), the main inhibitor of caspase-8 [43]. Viral and cellular dominant negative homologues of caspase-8 and caspase-10, coined v-FLIPs and c-FLIPs, respectively, have been discovered in the late 1990s [43,44]. Like caspase-8 and caspase-10 these inhibitors harbor two DEDs and as such, can be recruited within the TRAIL DISC (For recent reviews see, [41,45]). The long form of c-FLIP contains a caspase-like domain but this domain is devoid of caspase catalytic activity [43]. Owing to their ability to be co-recruited with caspase-8 within the DISC and to inhibit caspase-8 chain formation [46], self-processing and activation [47], c-FLIP isoforms are mostly associated with inhibition of apoptosis induced by ligands of the TNF family (Figure 3). It should be noted, however, that the long isoform of c-FLIP is also able to activate caspase-8 within the DISC [48,49] by mere dimerization in the absence of proteolytic activity [50]. However, activation of caspase-8 by this long isoform of c-FLIP does not lead to caspase-8 release to the cytosol and, thus, caspase-3 activation. Cells expressing high levels of this isoform are, thus, protected from apoptosis induced by ligands of the TNF family. Furthermore, increasing evidence suggests that c-FLIP isoforms can also inhibit other cell death machineries, including apoptosis induced by TLR3 [51], unfolded protein response [52], or chemotherapy [53,54,55].

Further downstream, in type II cells, since apoptosis requires the amplification of the caspase cascade through mitochondria ([56], see also Figure 1), resistance can also be induced by a deficiency in Bax [57] or the overexpression of B-cell lymphoma 2 (Bcl-2) anti-apoptotic protein family members, including Bcl-2 itself, Mcl-1, or Bc-xL (Figure 3) [58,59,60]. Last, but not least, irrespective of whether the tumor cell requires amplification of the signal through mitochondria or not, apoptosis induced by TRAIL can also be restrained by the overexpression of XIAP or survivin [61,62,63] (Figure 3).

Owing to the large number of events that can affect TRAIL’s efficacy, combined treatments associating cytotoxic drugs and TRAIL or TRAIL variants have emerged to restore TRAIL sensitivity in resistant tumor cells [24,64,65,66,67,68,69,70,71,72,73]. Interestingly, conventional or non-conventional chemotherapeutic drugs can overcome TRAIL resistance through (1) restoration of TRAIL agonist receptor expression [74,75,76,77,78,79,80]; (2) restoration of caspase-8 recruitment and activation at the DISC level [24,81,82], or inhibition of c-FLIP expression [66,83,84,85,86,87,88,89]; (3) inhibition of Bcl-2, Bcl-xL, Mcl-1, or up-regulation of Bax expression [60,65,90,91]; and/or (4) down-regulation of survivin and/or XIAP expression [61,65,92,93,94] (Figure 3).

### 2.3. Survival Signaling Pathways Regulating TRAIL-Induced Apoptosis

Signaling pathways such as mitogen-activated protein kinase (MAPK) and nuclear factor kappaB (NF-κB) were also reported to have a significant impact on the resistance or sensitivity of cancer cells to TRAIL [95]. TRAIL, itself, has been described to be able to induce their activation [96,97,98,99,100,101,102]. While it has been proposed that activation MAPK signaling by TRAIL involved the formation of a soluble secondary complex [103], the molecular events leading to their activation remain poorly understood.

In higher organisms, MAP kinases are a highly conserved family of protein kinases mainly consisting of three distinct members of MAPK, extracellular signal-regulated kinases (ERKs), c-jun N-terminal protein kinases (JNKs), and p38 MAP kinases [104]. MAPK-induced survival signaling cascades are impeded in several cellular events like cell differentiation, proliferation, inflammatory response, stress response, and cell death. These cascades are initiated by extracellular stimuli including, cytokines, growth factors, hormones, and environmental stressors, which transmit via transmembrane receptors into the intracellular compartment [105,106].

ERK1/2 activation was first associated with protection of transformed cells to TRAIL-induced apoptosis [99,107,108], but increasing evidence suggest that ERK activation could induce, through the transcription factor CHOP/GADD153, the up-regulation of DR5, as well as, to a lesser extent, that of DR4, allowing restoration of TRAIL-induced cell death in resistant cells [109,110,111,112,113,114,115,116]. JNK is involved in both cell proliferation and apoptosis, whereas p38 kinases are considered as cytokines regulating enzymes that are expressed in response to different stressful environmental conditions. The JNK pathway is regulated by the mitogen-activated protein kinase kinase (MKK) family proteins’-induced phosphorylation and is induced by TRAIL through the receptor-interacting protein kinase 1 (RIPK1) and TNF receptor-associated factor 2 (TRAF2) [117,118,119]. The mechanism of TRAIL-induced apoptosis linked to the activation of JNK and p38 kinase has been reported by several studies [73,91,120].

Similar to ERK, activation of JNK or p38, depending on the tumor cell line and trigger, can act in a transcriptional-dependent or independent manner to induce the regulation of TRAIL agonist receptors, c-FLIP or Bcl-2 family proteins to restore or inhibit apoptosis-induced by TRAIL [91,109,121,122,123,124,125,126,127,128,129,130,131,132,133].

The phosphoinositide-3-kinase–protein kinase B/Akt (PI3K-PKB/Akt) is a highly conserved, and tightly controlled, signaling pathway [134]. Its activation can be triggered by a wide range of stimuli, including tyrosine kinase receptors, integrins, B and T cell receptors, or G-protein-coupled receptors [135]. Activation of this survival pathway leads to the phosphorylation and activation of Akt by PI3K. Active Akt, in turn, phosphorylates a number of substrates [136], leading to transcriptional up-regulation or phosphorylation-dependent stabilization of survival genes such as c-FLIP, Bcl-2, Mcl-1, or XIAP, and ultimately to TRAIL resistance [137,138,139,140,141,142,143,144].

NF-κB is a nuclear transcription factor, expressed in response to a wide variety of stimuli such as inflammatory response, immune modulation, cell proliferation, and apoptosis. It consists of five subunits known as REL proteins, NF-κB1 (p50), NF-κB2 (p52), RelA (p65), RelB, and c-Rel (Rel) [145]. Normally, NF-κB exists in the cytoplasm in an inactive form by forming a heterodimer with its regulatory protein inhibitor IĸB. Phosphorylation of IĸB by IκB kinase (IKK) complex (IKKα and IKKβ) and its regulatory subunit NF-κB essential modulator (NEMO)/inhibitor of nuclear factor kappa-B kinase subunit gamma (IKKγ) releases NF-κB stimulating its translocation into the nucleus. Activation of NF-κB occurs via the molecular assembly of its subunits into homodimeric and heterodimeric forms that interact with the promoter of target genes [146].

Two independent signaling pathways were reported through which NF-κB signal transduction is activated [147,148]. The first pathway, the canonical pathway, is initiated by various stimuli, including tumor necrosis factor α (TNFα), interleukins-1β (IL-1β), interleukin-6 (IL-6), and members of toll-like receptors (TLRs) family. Binding of these ligands to their cognate receptors leads to the activation of the IKK complex and its regulatory subunit NEMO/IKKγ, inducing phosphorylation of IκBα on Ser32 and Ser36 residues [149,150]. Phosphorylation of IκBα on these serine residues enables the binding of an E3 ubiquitin protein ligase called beta-transducin repeat containing protein (β-TrCP), ubiquitination and proteosomal degradation of IκBα, leading to the release of NF-κB and to its nuclear translocation [151]. The non-canonical pathway is mediated by a group of non-inflammatory signals such as B-cell activation factor (BAFF)/CD40 essential for the survival of B-cell, the lymph node development factor lymphotoxin β (LTβ), and the receptor activator of NF-κB ligand (RANKL), which plays a central role in the differentiation of the osteoclasts [152]. Initiation of this pathway requires NF-κB-inducing kinase (NIK) but not the γ subunit of IKK, NEMO [153]. The p100 subunit of NF-κB linked to RelB is then phosphorylated by the activated IKKα and the phosphorylated p100-RelB complex is finally cleaved and processed, leading to the formation of the p52-RelB complex [154]. Activation of NF-κB canonical pathway has mostly been demonstrated to inhibit TRAIL-induced apoptosis through up-regulation of c-FLIP [96,98,119,155,156,157,158,159,160,161,162,163,164,165], but a recent study demonstrated that its activation in glioblastoma cells could contribute to Fas/CD95- and TRAIL-induced apoptosis [166].

## 3. Marine-Derived Compounds Regulating Apoptotic Signal Transduction Induced by TRAIL

**Figure 4:**
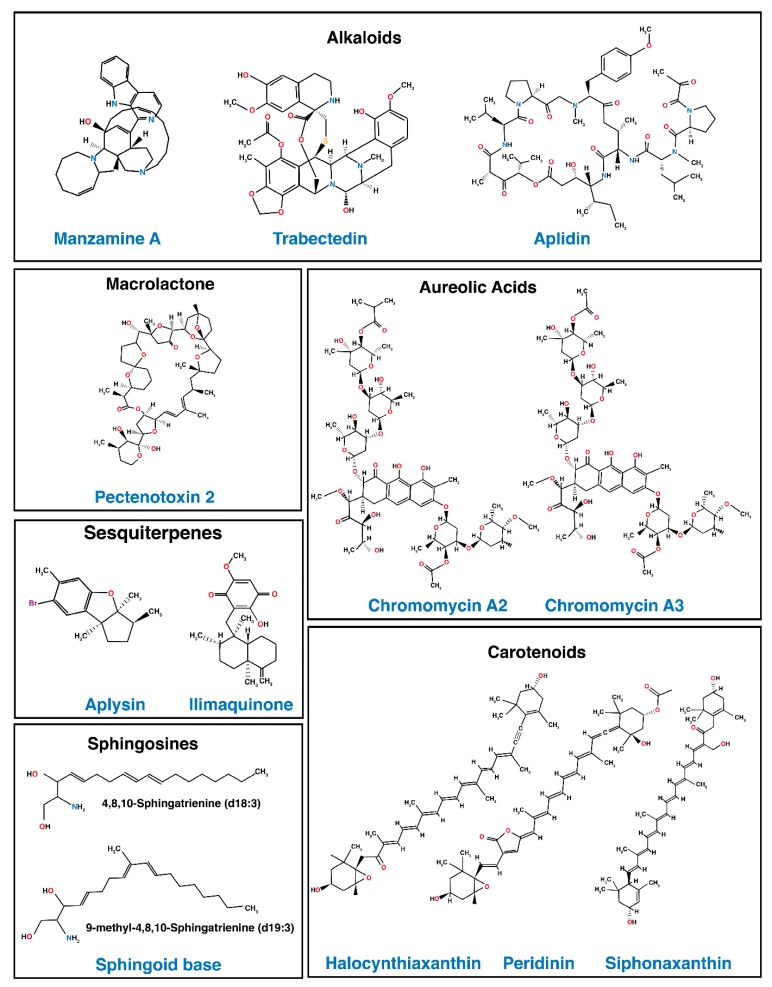
Selected marine-derived natural products with potent TRAIL-resistance overcoming activity.

Restoration or enhancement of apoptosis induced by TRAIL in resistant cancer is a prerequisite for the use of TRAIL in the clinic [167,168]. Exploration of natural products derived from terrestrial plants and marine fauna or flora pharmacopeia is, thus, likely to provide potent and selective TRAIL-sensitizing compounds [169,170,171,172]. The marine environment is enriched with a wide variety of living organisms, including bacteria, actinobacteria, sponges, fungi, microalgae, soft corals, seaweeds and flowering plants, such as mangroves, which have been reported as unexplored source for the discovery of potential anti-cancer compounds [173], as well as anti-bacterial, anti-fungal, antioxidants, or immunomodulatory activities [173,174,175]. This biomass diversity is considered as an unlimited source of structurally novel bioactive compounds, whose structural novelties, as well as variation in chemical features, confer to these organisms unique properties allowing them to survive in extreme marine environmental conditions, such as high salinity, temperature, or pressure [176,177,178]. Several natural marine products have been reported for their potent TRAIL-sensitizing effect in various TRAIL-resistant cancer cell lines (Table 1 and Figure 4) [171,179].

**Table 1:** Marine derived compounds with TRAIL-overcoming resistance activity and/or able to regulate TRAIL signaling components or inhibitors.

Chemical Class	Drug	Marine Source	Target Cell Line	Effects	Restores or Enhances TRAIL-Induced Apoptosis
Alkaloid	Manzamine A	Marine Sponge	AsPC-1 [180]	Inhibition of GSK3β and NF-κB	Yes
	Trabectedin	Marine Tunicate	MCF-7 & MDA-MB-453 [181]	Up-regulation of DR4, DR5, FADD, Bax, Bad Down-regulation of Bcl-2, Bcl-xL, XIAP and survivin	Yes
	Aplidin	Marine Tunicate	Multiple Myeloma cell lines and tumors [179]	Activation of p38 and JNK MAPK Down regulation of Mcl-1, Survivin Up-regulation of TRAIL	Not Tested
Aureolic acid	Chromomycins A2 Chromomycins A3	Actinomycetes	AGS [182]	Inhibition of wnt/β-catenin	Yes
Carotenoids	Halocynthiaxanthin	Oysters and Sea Squirts	DLD-1 [183]	Up-regulation of DR5	Yes
	Peridinin	Squirt Botrylloides	DLD-1 [183]	Up-regulation of DR5	Yes
	Siphonaxanthin	Green Algae	HL-60 [184]	Up-regulation of DR5 Down-regulation of Bcl-2	Not Tested
Macroloactones	Pectenotoxin-2	Marine Sponge	Hep3B & HepG2 [185]	Up-regulation of DR4, DR5, and Bax. Down-regulation of Bcl-2 and Bcl-xL	Not Tested
Sesquiterpene	Aplysin	Seaweed	MCF-7 [122]	Inhibition of p38 MAPK/ survivin pathway.	Yes
	Ilimaquinone	Marine Sponge	HCT116 & HT29 [126]	Up-regulation of DR4 and DR5 through ERK/p38 and CHOP	Yes
Sphingosine	Sphingoid bases	Sea Cucumber	HepG2 [186]	Up-regulation DR5 and Bax, Down-regulation of Akt/(PKB).	Not Tested

### 3.1. Marine-Derived Compounds Enhancing Apoptotic Signal Transduction Induced by TRAIL

Manzamine A is an alkaloid isolated from marine sponge *Haliclona* sp. displaying TRAIL-sensitizing activities. Manzamine A was suggested to restore TRAIL-induced apoptotic cell death in the TRAIL-resistance pancreatic AsPC-1 cell line via inhibition of glycogen synthase kinase-3 β (GSK3β) and subsequent inhibition of the survival factor NF-κB [180].

The marine-derived compounds chromomycins A2 and A3, extracted from actinomycete, are members of the aureolic acid family. These compounds have been shown to enhance human gastric adenocarcinoma AGS cell line sensitivity to TRAIL-induced cell death. The molecular mechanism involved remains unknown, but the authors suggested that sensitization could be correlated with chromomycin A2 and A3’s ability to inhibit wnt/β-catenin signaling [182].

Marine derived carotenoids have also been demonstrated to increase TRAIL sensitivity. Halocynthiaxanthin and peridinin, two carotenoids extracted from oysters and sea squirts were shown to display significant TRAIL sensitizing properties in the resistance colon cancer DLD-1 cell line [183]. Both carotenoids induced the up-regulation of DR5 and enhanced apoptosis induced by TRAIL.

Aplysin is a brominated sesquiterpene marine natural product isolated from the seaweed *Laurencia tristicha*. It was found to enhance apoptosis-induced by TRAIL in two human TRAIL resistant tumors, namely the MCF-7 breast cancer and A549 non-small lung cancer cell lines [122]. Restoration of TRAIL anti-tumor activity in these cells was proposed to occur through the activation of p38 MAPK and inhibition of survivin. Ilimaquinone was originally isolated from the Hawaiian sponge *Hippiospongia metachromia*. This sesquiterpene sensitizes human colon cancer cells to TRAIL through a CHOP-dependent regulation of DR5 [126]. In addition to DR5, up-regulation of DR4 and simultaneous down-regulation of Bcl-2 and Bcl-xL were also described in this study. Importantly, it was found that ilimaquinone-mediated up-regulation of both DR4 and DR5 required activation of ERK and p38 MAPK signaling as well as ROS production.

### 3.2. Marine-Derived Compounds Regulating TRAIL Signaling Components

Other marine-derived compounds have been described to exhibit antitumor properties associated with abilities to induce or repress expression levels of components of TRAIL death signal machinery. Likewise aplidin and trabectedin are two alkaloids from marine tunicate. Aplidin was demonstrated to induce the activation of p38 and JNK, to inhibit expression levels of Mcl-1 and survivin and to induce the expression of TRAIL in multiple myeloma primary tumors and cell lines [179]. In a previous study, aplidin was also found to induce translocation of TNF receptors into lipid rafts, in the leukemic Jurkat cell line, including translocation of DR5 [187]. Trabectedin, on the other hand, was suggested to induce apoptosis in resistant breast cancer cell lines either through the up-regulation of DR4, DR5, and FADD and the down-regulation of XIAP and survivin or through the up-regulation of Bax and Bak and the down-regulation of Bcl-2, Bcl-xL and survivin, in MCF7 and MDA-MB-453, respectively [181]. It should thus be kept in mind that since trabectedin is able to sensitize tumor cells to CD95/Fas-mediated cell death at a nM range [188], although it has not been described so far, this compound is thus also likely to restore apoptosis induced by TRAIL. Aplidin and trabectedin, are extensively assessed in phase II and III clinical trials for their antitumoral properties [189,190]. Of note, trabectedin, also known as Yondelis, has recently been approved by the FDA for patients suffering from advanced soft-tissue sarcomas [191].

In the same vein it is interesting to note that another carotenoid derived from green algae, siphonaxanthin, which exerts a potent cytotoxic effect on the human leukemia HL-60 cell line, was demonstrated to be able to induce the up-regulation of DR5 [184]. Although TRAIL sensitivity was not evaluated in this work, these findings suggest that this class of marine-derived carotenoids may be of interest to restore apoptosis induced by TRAIL (Table 1 and Figure 4).

Other marine derived compounds share the ability to induce the up-regulation of DR5 or to inhibit Bcl-2 anti-apoptotic members. Likewise, pectinotoxin-2 (PTX-2), a marine sponge-derived macroloactone, was shown to induce apoptosis in the human hepatocellular carcinoma Hep3B cell line. In these cells apoptosis correlated with the up-regulation of DR4, DR5 and Bax and the down-regulation of Bcl-2 and Bcl-xL [185]. Last, sphingoid bases extracted from sea cucumber were found to induce apoptosis the human hepatocellular carcinoma HepG2 cell line through a mechanism associated with inhibition of AKT phosphorylation and up-regulation of both Bax and DR5 [186].

## 4. Conclusions

Marine-derived compounds found, so far, to regulate directly or indirectly TRAIL-signaling represent only the tip of the iceberg. Through their ability to regulate MAPKs, NF-κB, ROS production, and ER stress (*i.e.*, GADD153/CHOP), these compounds are able to restore or induce the expression of TRAIL signaling pro-apoptotic partners and to reduce the amount of anti-apoptotic regulators. The extraordinary diversity of chemical structures naturally produced from ocean living organisms is, thus, more than likely to deliver potent molecules allowing the efficient use of TRAIL or TRAIL derivatives in the clinic. Twenty years have passed since the cloning of TRAIL [192]. Rendezvous in a decade or two to find out whether marine-derived compounds meet our expectations.
